# Signature changes in the expressions of protein-coding genes, lncRNAs, and repeat elements in early and late cellular senescence

**DOI:** 10.3906/biy-2005-21

**Published:** 2020-12-14

**Authors:** Gökhan KARAKÜLAH, Cihangir YANDIM

**Affiliations:** 1 İzmir Biomedicine and Genome Center, İzmir Turkey; 2 İzmir International Biomedicine and Genome Institute, Dokuz Eylül University, İzmir Turkey; 3 Department of Genetics and Bioengineering, Faculty of Engineering, İzmir University of Economics, İzmir Turkey

**Keywords:** Senescence, senescence-associated-secretory phenotype, lncRNA, repetitive DNA, repeatome, repeat elements, bioinformatics, genomics, transcriptome, RNA-seq

## Abstract

Replicative cellular senescence is the main cause of aging. It is important to note that early senescence is linked to tissue regeneration, whereas late senescence is known to trigger a chronically inflammatory phenotype. Despite the presence of various genome-wide studies, there is a lack of information on distinguishing early and late senescent phenotypes at the transcriptome level. Particularly, the changes in the noncoding RNA portion of the aging cell have not been fully elucidated. By utilising RNA sequencing data of fibroblasts, hereby, we are not only reporting changes in gene expression profiles and relevant biological processes in the early and late senescent phenotypes but also presenting significant differences in the expressions of many unravelled long noncoding RNAs (lncRNAs) and transcripts arisen from repetitive DNA. Our results indicate that, in addition to previously reported L1 elements, various LTR and DNA transposons, as well as members of the classical satellites including HSAT5 and α-satellites (ALR/Alpha), are expressed at higher levels in late senescence. Moreover, we revealed finer links between the expression levels of repeats with the genes located near them and known to be involved in cell cycle and senescence. Noncoding elements reported here provide a new perspective to be explored in further experimental studies.

## 1. Introduction

Aging is a natural process that is associated with many health issues, including—but not limited to—cardiovascular problems, neurodegenerative disorders, diabetes, liver disease, and cancer (Lopez-Otin et al., 2013). Also, the decline of proper immune function with age was reported against infections, including the infection caused by the novel coronavirus SARS-Cov-2 (Nikolich-Zugich et al., 2020). Even though the decline of functional tissue with age could be associated with the exposure to many external drivers such as inflammatory or genotoxic factors, cell division itself emerges as the main natural cause of aging. Many rounds of DNA replication result in an inevitable erosion of the telomeric ends of the chromosomes, triggering a DNA damage response that involves the p53 pathway, forcing the cell to exit the cell cycle and become senescent (d’Adda di Fagagna, 2008; Herranz and Gil, 2018).

Cellular senescence is characterised by distinct changes in the cell’s cytological and nuclear architecture, as well as the factors that it is releasing to its environment. Senescent cells are known to gain a senescence-associated-secretory phenotype (SASP), which involves various interleukins and interferons, giving rise to chronic inflammation in the aged tissues (Coppe et al., 2008). This inflammatory phenotype was shown to be highly influential on the onset of age-related diseases such as cancer and was undoubtedly linked to senescence-specific arrangements at the chromatin level. Importantly, the content and distribution of heterochromatin become altered along the way towards senescence, and senescence-associated-heterochromatic foci (SAHF) appear (Zhang et al., 2005). Notably, SASP and SAHF do not present themselves right after the cell exits its cycle, and senescence is known to be a gradual process (McHugh and Gil, 2018).

Several studies have attempted to identify senescence-specific genes to shed light on the universal programming events in the cell towards the onset of full senescence. Using several in vitro senescence cell models, such studies highlighted the downregulation of cell cycle genes, upregulation of inflammation-related genes, and the genes involved in the biogenesis of extracellular matrix, as well as secretory network (Purcell et al., 2014; Marthandan et al., 2015; u2001; u2002; u2003). In line with the drastic changes in gene expression profiles, the chromatin landscape is also transformed in the senescent cell. The methylation levels of certain histone residues (i.e. H3K4, H3K27, and H3K9) and the dysregulation in the activities of histone deacetylases (i.e. sirtuins) were found to be particularly important in promoting the senescent chromatin, which is associated with SAHF and the disorganisation of topological chromatin regions, including lamin-associated domains (LADs) (Criscione et al., 2016). In addition to protein-coding genes, long noncoding RNAs (lncRNAs) were also implicated in senescence (Puvvula, 2019). The functions of various lncRNAs, including HOTAIR, (Ozes et al., 2016), PANDA (Puvvula et al., 2014), MALAT-1 (Lei et al., 2017), and GUARDIN (Hu et al., 2018), were found to be particularly influential in maintaining the balance between cell proliferation and senescence. In addition to these, various novel lncRNA genes were also identified in a fibroblast senescence model (Abdelmohsen et al., 2013).

Nevertheless, the noncoding portion of RNAs emerging during senescence are not only limited to lncRNAs. MicroRNAs (Suh, 2018) and repetitive DNA transcripts (Criscione et al., 2016) were also reported. There are more than a thousand types of repeat motifs identified in the human genome, altogether comprising more than 50% of our DNA (Lander et al., 2001; de Koning et al., 2011). These elements, which are formed by tandem or interspersed repeat motifs and were originated from replication errors or ancient virus infections throughout our evolution (Jurka et al., 2007), are particularly important in maintaining a healthy chromatin architecture. Among such elements are the satellites, DNA and LTR transposons, and LINE and SINE elements. Their expressions are tightly regulated and have been implicated in human embryonic development (Probst et al., 2010; Yandım and Karakülah, 2019a) and were additionally linked to proteasomal activity (Natisvili et al., 2016). Moreover, their aberrant expression was linked to cancer (Ting et al., 2011; Yandım and Karakülah, 2019b). The specific chromatin marks associated with these elements are thought to be the key to their participation in genomic regulation (Pehrsson et al., 2019). HSATII and α-satellites, whose heterochromatinisation is essential for functional centromeres and kinetochores (Vos et al., 2006), were shown to become structurally extended in senescent cells (Swanson et al., 2013). HSATII, which is also overexpressed in epithelial origin cancers (Ting et al., 2011), was reported to be upregulated in senescent cells (De Cecco et al., 2013). Moreover, the activities of transposons were shown to be relevant in senescence. The L1 family of the long interspersed nuclear elements (LINEs) is activated both in mouse and human replicative senescence (De Cecco et al., 2013a; De Cecco et al., 2013b; u2008). In terms of long terminal repeats (LTRs), only MusD was reported in an aging mouse (De Cecco et al., 2013-b), and whether any members of the LTR family contribute to this phenomenon in humans has not yet been studied.

The studies mentioned above provided invaluable information on the transcriptome and chromatin signatures of cellular senescence in various models; however, there is still a paucity of knowledge on the differences between early and late cellular senescence, particularly at the level of lncRNA and repetitive DNA transcripts. It is important to understand the dynamics of the cell during the transition from early to late (deep) senescence because the outcomes of the two are vitally different. Early senescence is associated with wound repair and tissue regeneration (Demaria et al., 2014), whereas late senescence promotes a chronically inflammatory phenotype that is linked to fibrosis (Schafer et al., 2017) and cancer (Coppe et al., 2010). Moreover, there has been no holistic genome-wide study that reports the transcripts stemming from the complete repeatome during senescence despite the efforts to study invidiual repeats (e.g., L1s).

In this study, by employing a previously published (De Cecco et al., 2019) dataset (GSE109700) containing RNA-sequencing (RNA-seq) data from biological triplicates of early proliferating, early replicative senescent, and late replicative senescent LF1 human embryonic lung fibroblast cells with at least 70 million reads, we have quantified transcripts from protein-coding genes, lncRNAs, and repeat elements. We have also distinguished the transcriptomic signatures of early and late senescent cellular phenotypes, providing the full noncoding portion of the senescent transcriptome. The dataset we used was generated with the aim of detecting L1 transcripts and is therefore suitable for the quantification of transcripts from protein-coding genes and lncRNA genes, as well as all types of repetitive DNA at the same time. We were also able to confirm our key findings on an independent RNA-seq data (GSE63577) obtained from MRC5 fibroblasts (Marthandan et al., 2016). Our results highlight distinct biological processes and stage-specific lncRNAs, as well as previously unspecified repeat transcripts in the comparison of early and late senescent phenotypes versus the young and proliferating phase.

## 2. Materials and methods

### 2.1. Data collection and processing

Sequencing reads of nine samples, including biological triplicates of cells that are in the early proliferating state and those that are in early and late replicative senescence, respectively, were downloaded from the Sequence Read Archive database (Leinonen et al., 2011) (SRA Accessions: SRP131506 and SRP050179) with SRA Tool Kit v.2.9.0, using the following command: *“fastq-dump –gzip –skip-technical –readids –dumpbase –clip –split-3”* . Human reference genome GRCh38 and the associated gene annotation in GTF file format (Release 32) were downloaded from the GENCODE project web sitehttps://www.gencodegenes.org. Repeat annotations were collected from RepeatMaskerhttp://repeatmaster.org. RNA-seq datasets were aligned to the reference genome with the Rsubread v1.34.7 package (Liao et al., 2019) of R v3.5.1 statistical computing environmenthttps://www.r-project.org/ with the following settings: *“align(index={index file}, readfile1={input_1.fastq}, readfile2={input 2.fastq} type=”rna”, input_format=”gzFASTQ”, output_format=”BAM”, output_file={output file}, nthreads=numParallelJobs)”* . We utilised SAMtools v1.3.1 (Li et al., 2009) in order to sort and index all BAM files generated in the alignment step.

For the measurement of expression levels of protein-coding and lncRNA genes, we employed the featureCounts function (Liao et al., 2014) of the Rsubread package with the following command: *“featureCounts (files = {infile.bam}, annot.ext = “{infile.gtf}”, isGTFAnnotationFile = T, GTF.featureType = “exon”, GTF.attrType = “gene_id”, useMetaFeatures = T, countMultiMappingReads = T, isPairedEnd = T, nthreads = numParallelJobs)”* . Again, we utilised the featureCounts function in the R environment to quantify repeat expressions. However, repeat element features overlapping exons of protein-coding and lncRNA genes were filtered out from the annotation file before the quantification steps in order to minimise the ambiguity caused by their repetitive nature. We considered only uniquely mapped reads aligned to DNA, LINE, SINE, LTR, and satellite repeat regions. Repeat element and gene counts were then merged into a single count matrix for further downstream analysis.

### 2.2. Differential expression and gene ontology enrichment analyses

Counts per million (CPM) values were calculated for protein-coding genes, lncRNAs, and repeat element families and subfamilies across all samples. We filtered out the features where expression levels  were CPM <1 in each condition and only considered samples where at least one condition has CPM >1 threshold in at least two replicates. To determine differentially expressed genomic features between two conditions, we made use of the EdgeR package v3.24.3 (Robinson et al., 2010). In this step, trimmed mean of M-values (TMM) normalisation was applied to the filtered count values, and the dispersions were estimated with estimateDisp function for each comparison. For the calculation of false discovery rate (FDR) of each feature, appropriate contrast statistics were employed using exactTest function of edgeR. Next, all features were sorted based on an absolute log2 fold change value between conditions, and top-50 genes, lncRNAs, and repeat elements were determined individually. Gene ontology (GO) analysis on the differentially expressed genes was performed using DAVID’s functional annotation tool (Huang da et al., 2009).

### 2.3. Identifying the potential effects of repeat elements on proximal gene expression

In order to identify statistically significant associations between repeat element expression and the transcription of adjacent genes across samples, we made use of the TEffectR package, which was recently developed by us (Karakülah et al., 2019). This tool takes sorted and indexed genome-aligned BAM files as input and predicts potential associations by establishing multiple linear regression models based on read count values of genes and repeats. We utilised the TEffectR package with default settings for DNA, LINE, SINE, LTR, and satellite repeat elements; the *“strand”* and *“distance”* parameters of the TEffectR::get_overlaps function were set as *“strandness”* and *“5000”* , respectively

### 2.4. Statistical analysis and graphical representation

R statistical computation environment was utilised for all statistical analyses. We employed pheatmap package of Rhttps://CRAN.R- project.org/package=pheatmap to draw all the heatmaps, on which expression values were represented in the rows. Clustering was performed with the Euclidian method, and the prcomp function was used for principal component analysis (PCA). Other graphics were obtained using the ggplot2 packagehttps://ggplot2.tidyverse.org/.

## 3. Results

### 3.1. Early and late senescence phenotypes differ in their transcriptomes globally

**Figure 1:**
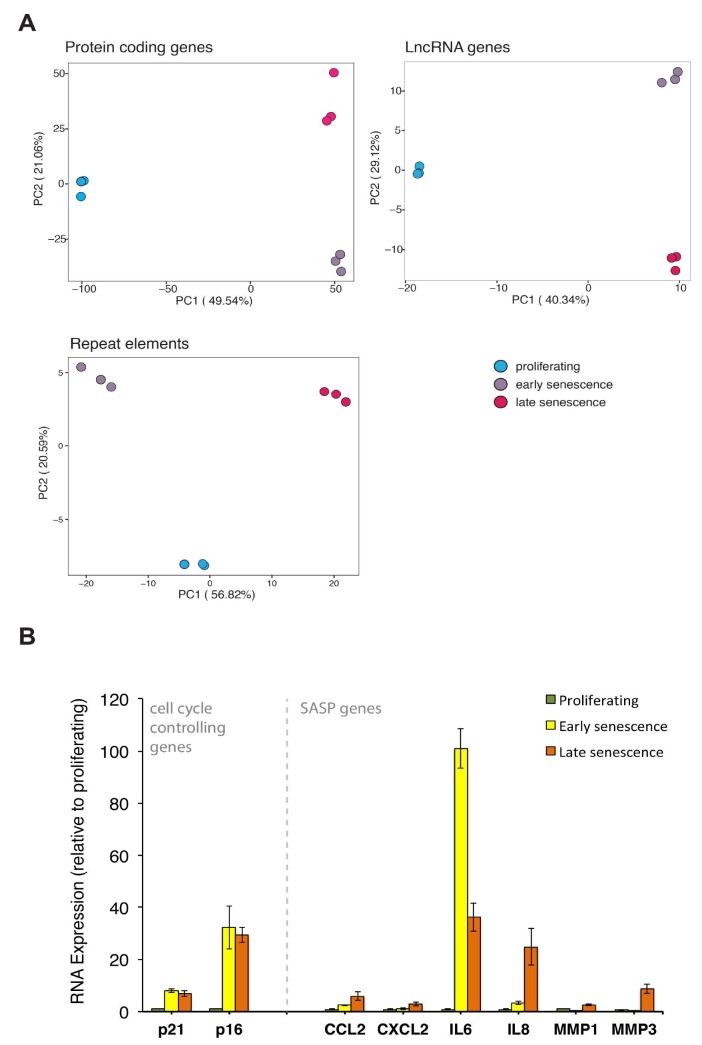
Global changes in the transcriptome of proliferating, early senescent, and late senescent LF1 fibroblasts. (A) principal component analysis (PCA) on the RNA expression levels of protein-coding genes, lncRNAs and repeat elements. (B) expression levels of cell cycle controlling and senescence-associated-secretory phenotype (SASP) related genes.

We analysed RNA-seq data from three biological replicates of young proliferating, early senescent, and late senescent LF1 human lung fibroblast cells (Supplementary Tables S1 and S2). The characterisation of their proliferating and senescent phenotypes at distinct early and late stages was previously done (De Cecco et al., 2019). Our PCA at the global transcriptome level showed that each biological group (i.e. proliferating, early senescent, and late senescent) clustered separately and that the triplicates were close to each other, indicating the high reliability of the dataset (Figure 1a). The separation of the clusters was prominent not only for protein-coding genes but also for lncRNA, as well as repetitive DNA transcripts, implicating the contribution of all of these elements to different phases of senescence. To confirm that our analysis is correct and in line with the previous publication (De Cecco et al., 2019), we also analysed the expression levels of cell cycle control genes p21 and p16 (Herranz and Gil, 2018) and genes that are known to be associated with SASP (Salama et al., 2014; De Cecco et al., 2019). The expression of the cell cycle inhibitors p21 and p16 increased in the senescent phenotypes, and the SASP genes were only expressed at a mild level in the early senescent phenotype and at a higher level in late senescence, with the exception of IL6, which seems to be expressed at a higher level in early senescence (Figure 1b). This agreed well with the previous publication (De Cecco et al., 2019).

**Figure 2:**
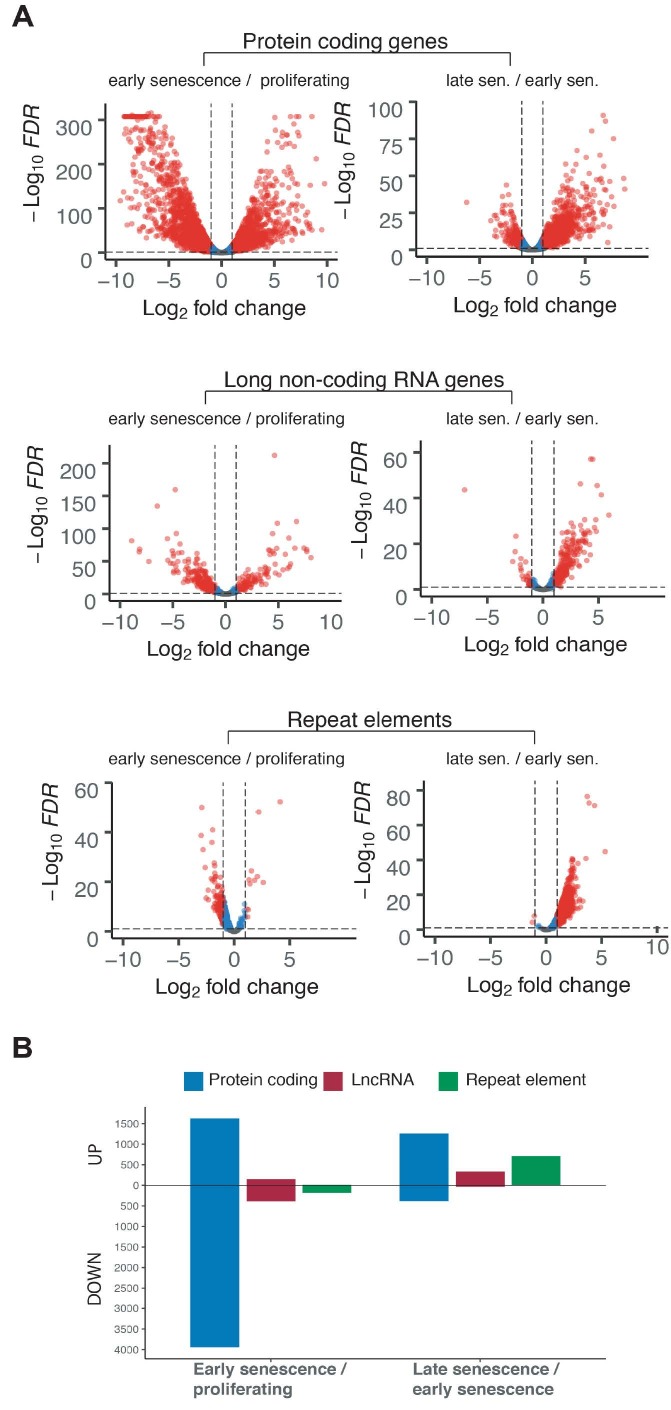
Differentially expressed transcripts arisen from protein-coding genes, lncRNA genes and repetitive DNA elements in early and late senescence in LF1 fibroblasts. (A) volcano plots summarising the changes in the transcripts in 3 different categories: protein-coding, lncRNA, and repeat element. (B) bar-plot illustrating the number of upregulated and downregulated transcripts in the transition from the proliferating state to early senescence and that from the early senescent stage to late senescence. (|log2(Fold Change)|>1 and FDR < 0.05).

We analysed differentially expressed protein-coding genes, lncRNAs, and repetitive DNA transcripts and visualised the changes in their transcription using volcano plots (Figure 2a). We realised that most of the changes in the protein-coding genes occurred when cells entered early senescence. Over 1500 genes were upregulated in the early senescent phenotype when compared to proliferating cells, and over 3500 of them were downregulated (|log2(Fold Change)|>1 and FDR < 0.05) (Figure 2b). There was an obvious reduction in the number of downregulated genes when cells in late senescence were compared to early senescent phenotype. Moreover, most of the changes on the lncRNA and repetitive DNA transcripts were on the downregulation side, comparing the proliferating phenotype to the early senescent one, whereas an upregulation was more prominent in the early to late senescence transition. Indeed, there seemed to be a global upregulation in repeat expression in the late senescent phenotype. Considering the importance of repetitive DNA in the formation of chromatin architecture and the fact that they are normally silenced, it is likely that the major chromatin changes in the late senescent phenotype harbour repeat elements.

### 3.2. Distinct biological processes in early and late senescence stages

**Figure 3:**
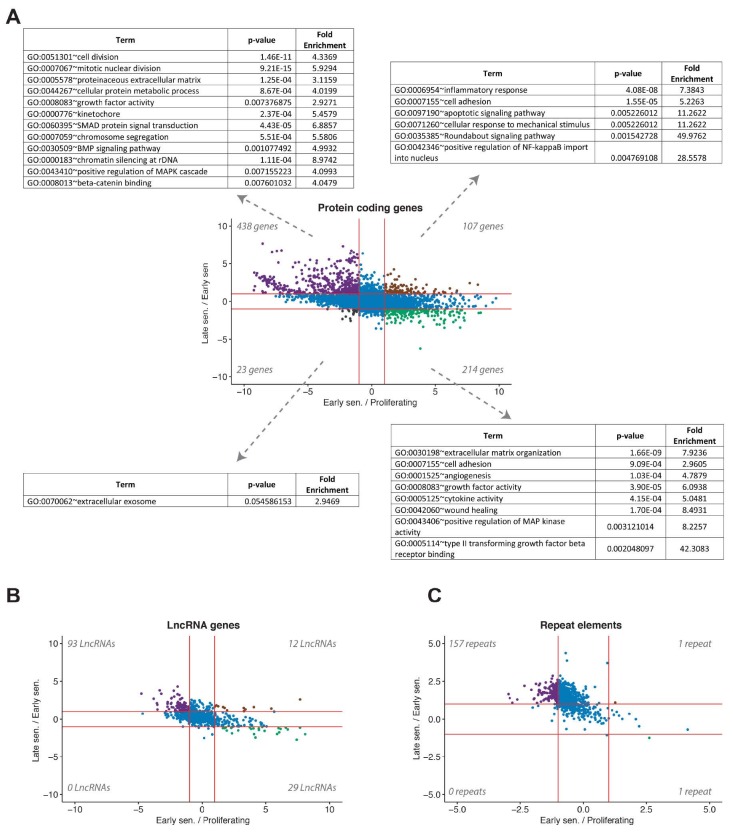
Differentially expressed RNAs in cross comparison with early senescence/proliferating and late senescence/early senescence in LF1 fibroblasts. (A) differentially expressed genes and their enrichment in (GO) terms. (B) differentially expressed lncRNAs. (C) differentially expressed repeat elements (|log2(Fold Change)|>1 and FDR < 0.05).

Biological pathways that take stage during the transitions towards early and late senescence are generally revealed. De Cecco et al. (2019) employed Gene Set Enrichment Analysis (GSEA) (Subramanian et al., 2005) and pointed out that cytokine and toll-like receptor signalling, retinol, and olfactory pathways were implicated at distinct senescence phases. They performed a paired comparison for each group separately. To better visualise the changes in gene signatures and employ a more holistic approach, we generated a scatter plot where the x-axis denoted differentially expressed genes (|log2(Fold Change)|>1 and FDR < 0.05) in early senescence when compared to proliferating cells, and the y-axis denoted differentially expressed genes in late senescence in comparison to early senescence (Figure 3a). Next, we performed a gene ontology analysis using DAVID (Huang da et al., 2009) (Supplementary Table S3). There were 438 genes upregulated during the transition from early to late senescence. These genes were intriguingly downregulated in the transition to early senescence from the proliferating state. Along these genes were those related to cell division and proliferation, along with some others involved in SMAD, BMP, and β-catenin signalling pathways. Moreover, 107 genes that are involved in inflammatory response, cell adhesion, apoptotic signalling, cellular response to mechanical stimuli, roundabout signalling, and NF-κB pathways were gradually increased as the progression towards late senescence continued (i.e. gradually upregulated both in early and late senescence). On the other hand, 214 genes linked to extracellular matrix organisation, angiogenesis, wound healing, MAPK, and TGF-β pathways were only upregulated in the transition from the proliferating state to early senescence. Finally, 23 genes associated with extracellular exosome were continuously downregulated in early and late senescence (i.e. gradually downregulated both in early and late senescence). When differentially expressed lncRNAs were analysed with similar filters, it was realised that some lncRNAs were only upregulated in the late phenotype and some others were only upregulated in the early phenotype (Figure 3b). Interestingly, almost all differentially expressed repeat elements were upregulated only during late senescence (Figure 3c), consistent with what is presented in Figure 2b.

### 3.3. Top-variable signature transcripts reveal stage-specific senescence markers in protein-coding genes, lncRNAs, and repeat-arisen transcripts

**Figure 4:**
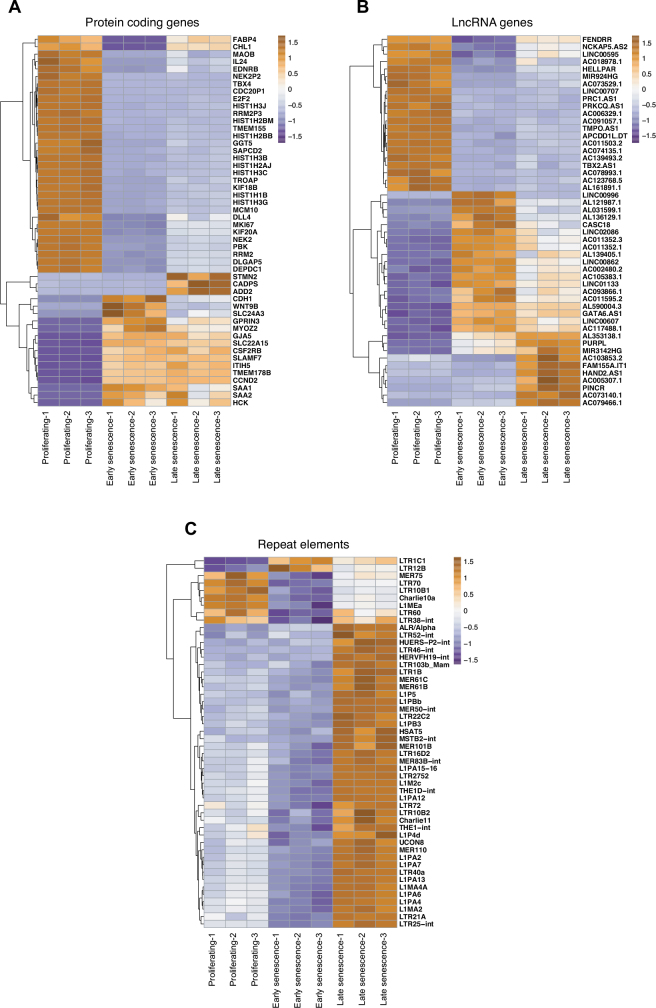
Heatmaps representing transcripts that ranked in the top 50 in terms of their expression variations in LF1 fibroblasts. Topvariable 50 transcripts in (A) protein-coding genes, (B) lncRNA genes, and (C) repeat elements.

We calculated the top-variable 50 transcripts for protein-coding genes, lncRNAs, and repeat transcripts and visualised these with heatmap representation (Figure 4). CDH1 (E-cadherin), WNT9B (a member of the classical Wnt family), SLC24A3 (a member of sodium/calcium exchangers), GPRIN3 (G protein inducer of neural outgrowth protein), and MYOZ2 (calcineurin binding sarcomeric muscle protein) genes were expressed at their highest levels in early senescent cells. On the other hand, STMN2 (a member of the stathmin family involved in neural growth), CADPS (calcium dependent secretion activator), and ADD2 (cytoskeletal beta adducin) genes were expressed highly in late senescent cells (Figure 4a). Moreover, we identified 18 lncRNAs to be expressed particularly highly in proliferating cells, 19 lncRNAs in early senescent cells, and 10 others in late senescent cells (Figure 4b). Most of these lncRNAs were novel and not previously addressed in the literature on senescence and aging. When we checked the repetitive DNA transcripts, MER75 (DNA transposon), LTR70 (LTR transposon), LTR10B1 (LTR transposon), Charlie10 (DNA transposon), and L1MEa (LINE transposon) were expressed at their highest in proliferating cells (Figure 4c). LTR1C1 and LTR12B (LTR transposons) were expressed highly in early senescent cells and most of the other top-variable repeats were expressed at their highest level in late senescent cells. The latter included members of the previously presented L1 (LINE) family (De Cecco et al., 2019), α and HSAT5 satellites, as well as various members of the DNA and LTR transposons family. Our results on repeat transcripts indicate that repeat expression in late senescence is a broader phenomenon than previously described by De Cecco et al. (2019), with many previously unmentioned elements being dysregulated.

### 3.4. Significant associations between repetitive DNA motifs and the regulation of nearby genes related to cell cycle, senescence, and aging

Repeat motifs on DNA are known to modulate the expressions of proximal genes (Huda et al., 2009; Elbarbary et al., 2016). To see if repeats might have a potential link with the expression statuses of nearby genes in the dataset we utilised, we employed TEffectR, a bioinformatics tool that predicts the associations between the expressions of the uniquely positioned repeats that are present in the proximity of genes using a linear regression model (Karakülah et al., 2019). We checked the repeats uniquely positioned within the 5kb upstream region of genes. Out of the significant repeat/gene associations, at least a dozen comprised genes that are involved in cell cycle, senescence, or aging. For example, a uniquely positioned LINE element L2b could explain 77.21% (P = 0.001) of the expression of its proximal gene FOXM1, which is a master regulator of the cell cycle (Smirnov et al., 2016). Another LINE element L1PA13 could explain 66.66% (P = 0.004) of the expression of its proximal gene NFKBIZ, which is known to promote SASP (Alexander et al., 2013). The full list of relevant associations is presented in Table. These significant associations, which are worth exploring experimentally, could potentially imply the contribution of repeats to the genomic regulation of cellular senescence.

**Table:** Significant links between the expressions of genes and that of uniquely positioned repeat elements within the 5kb promoter regions in LF1 fibroblasts. TEffectR, a tool that utilises a linear regression model to uncover significant links between repeat expression and proximal gene expression, was employed to analyse associations between the expressions of repeats and the proximal genes involved in the cell cycle, senescence, and aging.

Gene name	Link to cell cycle, senescence and aging	Repeat name	R-square	Adj. R-square	Linear model P-value
FOXM1	A key transcription factor regulating cell cycle and senescence (Smirnov et al. 2016)	LINE: L2b	0.8006	0.7721	0.0011
NFKBIZ	Inhibitor of NFκB and co-activator of various NFκBtargets; an important regulator of SASP(Alexander et al. 2013)	LINE: L1PA13	0.7083	0.6666	0.0044
NPIPB3	A nuclear pore interacting protein involved in the aging of muscle (Willis et al. 2020)	LINE: L2	0.7589	0.7245	0.0022
PPIC	Immunophilin/isomerase involved in inflammatory response (Yamaguchi et al. 2011) and age-related cardiovascular disease(Alfonso et al. 2019)	LINE: L1ME3a	0.8519	0.8307	0.0004
SHB	Adaptor protein regulating cell cycle in hematopoietic stem cells(Gustafsson et al. 2013)	LINE: L2a and L2c	0.7961	0.7281	0.0085
SYNGR3	Synaptic vesicle protein contributing to synaptic dysfunction in the aged Alzheimer’s brain (Saetre et al. 2011)	LINE: L2	0.6717	0.6248	0.0069
EID3	A transcriptional co-repressor which was linked to senescence (Wang et al. 2018)	SINE: AluSp	0.9005	0.8862	0.0001
ICAM5	Cell adhesion protein regulating the expression of age-related genes (Crossland et al. 2017)	SINE: AluJr	0.8652	0.8460	0.0003
GSDMB	Contributes to inflammasome signaling associated with aging (Chen et al. 2019; Feng et al. 2018; Mejias et al. 2018)4)	SINE: AluSz6 and MIRb	0.9711	0.9615	0.00002
FER1L4	Encodes a long non-coding RNA that competes and regulates PTEN expression resulting in decreased cell growth (Xia et al. 2015)	DNA: Charlie4z and MER1B	0.9029	0.8705	0.0009
IGIP	Induces the expression of IgA, which is elevated with aging (Austin et al. 2003; McDonald et al. 2011)	DNA: Charlie4z	0.9704	0.9662	0.000001
NUAK1	A kinase that induces replicative senescence and linked to genomic instability (Humbert et al. 2010)	LTR: LTR7 and MLT1I	0.8369	0.7825	0.0043

### 3.5. Confirmation of key transcripts on an independent dataset

In order to verify our key findings, we employed another dataset (GSE63577), where fibroblasts were cultured until they reached the late stage of replicative senescence (Marthandan et al., 2016). The authors in this study performed RNA-seq on fibroblasts, which were at different levels of senescence as determined by a population-doubling (PD) graph. Among the fibroblasts that they used, the RNA collection points of MRC5 fibroblasts were the only ones that resembled proliferating, early, and late senescence phases observed in LF1 cells in the De Cecco et al. (2019) study, even though the biochemical characterisation of senescence was not done as extensively.

We quantified the transcripts and provided the count numbers and differential expression results in the Supplementary Tables S4 and S5. The PCA plot shows that the variation between triplicates was higher than the GSE109700 dataset (Supplementary Figure S1), and we detected that the majority of differentially expressed genes emerged in the early to late senescence transition (PD62 vs. PD72) in this dataset. Still, the majority of upregulation of lncRNAs and repeat transcripts took place in late senescence (Supplementary Figure S2), agreeing well with LF1 cells. The heatmaps (Supplementary Figure S3) showed that some key transcripts followed similar trends with what was observed in the LF1 cells.

**Figure 5:**
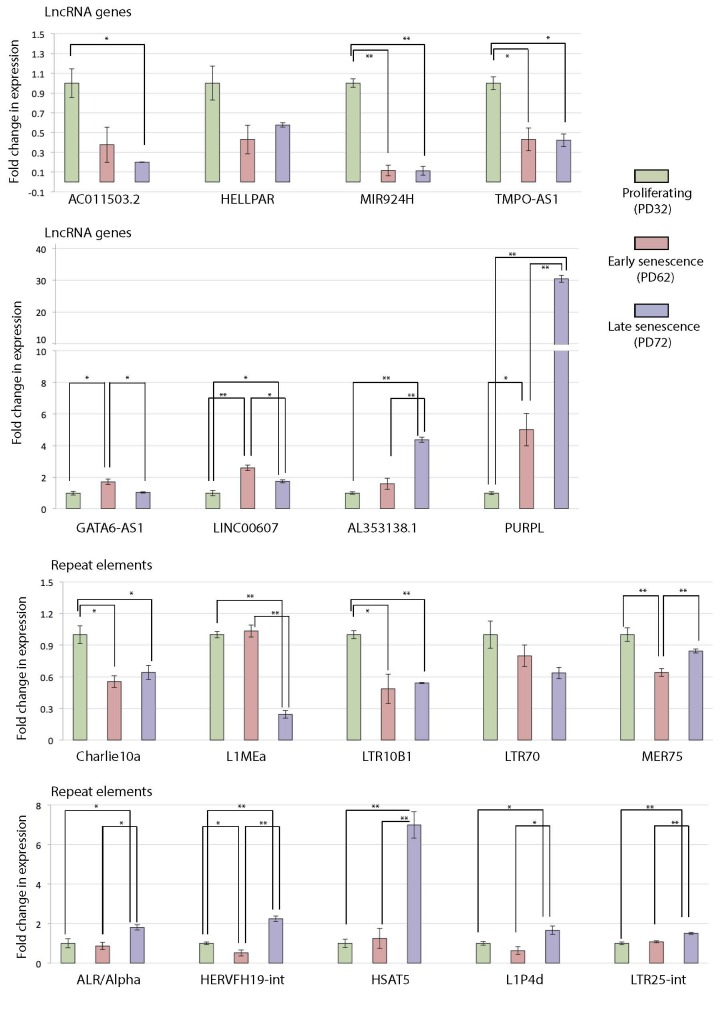
Confirmation of expression changes in lncRNAs and repeat elements in proliferating (PD32), early senescent (PD62), and late senescent (PD72) MRC5 fibroblasts by RNA-seq. A T-test was applied (*P < 0.05, ** P < 0.01). Fold changes relative to proliferating state (set to 1.0) were calculated directly from CPMs. Error bars represent standard error of the mean.

We were able to verify important transcripts and highlighted particular signature changes in the expressions of lncRNAs and repeat elements in the MRC5 fibroblast dataset (Figure 5 and Supplementary Table S5). Results were in line with the LF1 dataset (Figure 4 and Supplementary Table S2). PURPL expression is already known to increase as the cells proceed into senescence (Casella, et al. 2019), but LNC00607, AL353138.1 were not linked to senescence before. On the other hand, we are reporting that AC011503.2, HELLPAR, MIR924HG and TMPO-AS1 were expressed at the highest level in the proliferating state. Moreover, key repeats such as Charlie10a, L1MEa and LTR10B1 were linked with the young and proliferating state whereas classical satellites α- (ALR/Alpha) and HSAT5 as well as transposons HERVFH19-int and L1P4d were significantly linked to late cellular senescence. GO analysis of MRC5 senescence model revealed that cell proliferation, extracellular matrix, inflammatory response, cytokine activity, growth factor activity, cell-cell signalling, angiogenesis, MAPK pathway and wound healing were pronounced in agreement with LF1 cells (Figure 3 and Supplementary Table S6). However, the quarters (senescence stages) where these pathways appeared were not exactly same in comparison to LF1 cells.

Finally, we compared the expression correlations in MRC5 cells (not shown in Table) with the correlations obtained from LF1 cells and realised that correlations between L2 and NPIPB3 (R2 = 0.8284, P < 0.01); GSDMB and MIRB (R2 = 0.8959, P < 0.01); FER1L14; and MER1B (R2 = 0.7801, P < 0.05) were significant along with an insignificant trend seen between NFKBIZ and L1PA13 (R2 = 0.5129, P = 0.1589).

## 4. Discussion

The plastic nature of genomic expression and the progressing events of the regulatory mechanisms are essential to many contexts in biology. Replicative cellular senescence is perhaps one of the most fundamental phenomena occurring naturally at different paces in every living organism. The high number of diseases associated with age and the cellular senescence, including cancer and neurodegenerative disorders, and the fine-tuned balance in which early senescence is particularly adjusted for tissue regeneration and development, while late senescence is disease-inducing, bring about the need for revealing the finer details of this phenomenon. Our comparative and holistic analysis on proliferating cells and those that are in early and late replicative senescence confirms some of the previously published findings and sheds further light on the formerly unreported dimensions of genomic regulation towards senescence. Our results on the gene expression signatures of inflammatory response, cytokine activity, and cell adhesion agree well with De Cecco et al. (2019), but we also posit that SMAD, BMP, TGF-β, β-catenin, and NF-κB signalling pathways are involved at distinct stages of senescence using the same dataset. The exosome activity pathway seems to be continuously downregulated as cells proceed into senescence. More interestingly, pathways related to tissue regeneration such as wound healing and angiogenesis were only enriched in cells of early senescence but not in late senescence. Agreeing well with this, genes involved in growth factor and cytokine/inflammation activity appeared in different categories, suggesting their stage-specific expression in the early and late stages of senescence.

An interesting feature realised in this study was that most of the differential expressions of noncoding RNA transcripts were downregulated in early senescence and upregulated in late senescence (Figure 2b and Supplementary Figure S2). It is known that proper heterochromatin on repetitive DNA is maintained through human development, and this is established right after the first phase of preimplantation (u20fda). Early senescent cells comply well with this fact, perhaps sharing characteristics of the developmental period required during regeneration. Indeed, cell lineage differentiation needs a pause in the cell cycle (Kaldis and Richardson, 2012), and our results indicate that when cells first enter replicative senescence, they resemble this period in terms of the expression regulation of the noncoding portion; most of the noncoding RNAs (particularly repeats) were still repressed, but cell cycle was halted. Still, some lncRNAs (e.g., MIR924HG and TMPO-AS1) and particular repeats (e.g., Charlie10a, L1MEa, and LTR10B1) were linked to the proliferating state, and their functions are yet to be characterised for cell cycle regulation. On the other hand, given that unstable repeat transcripts were linked to genomic instability (Younger and Rinn, 2015), the relatively high level of repeat transcripts at deep/late senescence stage could be a potential factor manifesting cancerous events in such cells (Zhu et al., 2011; Kishikawa et al., 2016; u20ff; u2100; u2101). In addition to studies that linked HSATII and L1 (LINE transposon) expression to cellular senescence (u2102; u2103), our study states that α-satellites (ALR/Alpha) and HSAT5 are specifically expressed in late senescence. HSATII expression was below the 1 CPM threshold in our analysis but showed a slight upregulation that was not statistically significant in late senescence. According to the DFAM database (Hubley et al., 2016), most of the HSAT5 sequences are located at pericentromeres, which are essential components of cellular heterochromatin (Martens et al., 2005). Moreover, we report that previously presented L1 transcripts are not the only transposon-originated RNAs abundant in the senescent transcriptome and that the expressions of many LTR elements and DNA transposons also become activated. As opposed to L1s in general, L1MEa expression was at its highest in the proliferating state, and this emphasises the fact that not all members of the L1 family could be linked to senescence, which is different than previous perceptions in the field. We found that L1P4d is particularly expressed in late senescence. In addition, previously unmentioned LTR retrotransposons such as HERVFH19-int and LTR25-int expressions were also associated with late senescence. Even though no specific contributions were reported for these particular LTR transposons in the literature before, LTR activity has generally been linked to age-related diseases such as autoimmune disorders, neurological conditions, and cancer (Attig et al., 2019; Saleh et al., 2019).

We verified the above-mentioned lncRNAs and repeat transcripts in two independent datasets (GSE109700 and GSE63577) with the caveat that the second dataset (GSE63577) displayed a higher variation between triplicates. Also, the RNA sequencing library preparation methods of the two datasets were different. De Cecco et al. (GSE109700) provided a more extensive characterisation of the cells in displaying their proliferating, early, and late senescent features biochemically. Still, the verification of key noncoding transcripts at distinct stages of senescence in both datasets emphasises their emergence in the senescence phenomenon.

In conclusion, our report illuminates further dimensions of the transcriptomic changes linked to senescence and provides the distinguishing features of the early senescent cells in comparison to late senescence in terms of activated biological processes and noncoding transcripts. LncRNAs and repeat-arisen transcripts, most of which were reported in terms of their link to senescence for the first time here, could be investigated further to uncover the hidden complex mechanisms of senescence or serve as markers to predict cellular age. The effects of repetitive DNA expression could be rather global, but the significant links between the proximal genes we found also suggests that there are finer details at the individual gene level. Specific transcripts expressed at different stages of senescence and listed in this report could be useful for future studies investigating the age-related changes of the cellular microenvironment and genomic integrity affecting the behaviour of cancer with age. Also, future innovations in wound repair and tissue regeneration could benefit from the genes listed as specific for the early senescent phenotype.

## Supplementary data

Supplementary figures and tables can be downloaded from http://dx.doi.org/10.17632/839nknw58h.1.
